# Enriched environment improves working memory impairment of mice with traumatic brain injury by enhancing histone acetylation in the prefrontal cortex

**DOI:** 10.7717/peerj.6113

**Published:** 2018-12-07

**Authors:** Xin Wang, Zhaoxiang Meng, Jibing Wang, Hongyu Zhou, Yi Wu, Junfa Wu

**Affiliations:** 1 Department of Rehabilitation Medicine, Huashan Hospital, Fudan University, Shanghai, PR China; 2 Department of Rehabilitation Medicine, Clinical Medical College, Yangzhou University, Yangzhou, Jiangsu, PR China

**Keywords:** Traumatic brain injury, CREB binding protein (CBP), Acetylation homeostasis, Choline acetyltransferase, Working memory, Enriched environment

## Abstract

Working memory impairment is a common cognitive dysfunction after traumatic brain injury (TBI), which severely affects the quality of life of patients. Acetylcholine is a neurotransmitter which is closely related to cognitive functions. In addition, epigenetic modifications are also related to cognitive functions. A neurorehabilitation strategy, enriched environment (EE) intervention, has been widely used to improve cognitive impairment. However, studies of the mechanism of EE on cholinergic system and epigenetic modifications in mouse with TBI have not been reported yet. In this paper, a mouse model with traumatic frontal lobe injury was established, and the mechanism on EE for the mice with TBI was explored. It was found that EE could improve Y-maze performance of mice with TBI, the function of cholinergic system, and the imbalance of acetylation homeostasis in the prefrontal cortex of contralateral side of TBI. In addition, EE also could increase the level of CREB binding protein and histones H3 acetylation at ChAT gene promoter region in the prefrontal cortex of contralateral side of TBI. These indicate that EE has an important effect on the improvement of working memory impairment and the underlying mechanism may involve in histones H3 acetylation at ChAT gene promoter regions in the prefrontal cortex.

## Introduction

Traumatic brain injury (TBI) is commonly caused by increasing incidences of traffic and occupational accidents. It is currently a major public health problem, and is associated with high mortality and morbidity worldwide (Li et al., 2015; Mollayeva et al., 2016). People with TBI often develop cognitive deficits, such as attention deficit, working memory impairment and so on, which seriously affects the patients’ life quality (Dobryakova, Boukrina & Wylie, 2015).

Working memory is one of higher brain functions that enables us to hold information temporarily and manipulate the content online to execute complex cognitive tasks. The neural basis of working memory is theorized to depend on persistent firing of the prefrontal cortex (Hernandez et al., 2018). More and more studies also have shown that the prefrontal cortex is a key brain region for working memory (Hernandez et al., 2018; Cavanagh et al., 2018). However, there are few reports on the specific mechanisms between working memory impairment and damage to the prefrontal cortex after traumatic brain injury, as well as the related mechanisms involved in the improvement of work memory impairment.

Cognitive functions involve the participation of many neurotransmitters, especially acetylcholine (Ach) (Lombardo & Maskos, 2015; Wang et al., 2013). The Ach levels in patients with Alzheimer’s disease (AD), a disorder characterized by cognitive and memorial dysfunctions, are dramatically reduced in the brain (Lombardo & Maskos, 2015). In both patients and animal models of TBI, dysregulation of cholinergic system was found. Cognitive impairment induced by TBI could be improved by enhancing the levels of Ach in the brain (Shin & Dixon, 2015; Arciniegas, 2011). Some studies showed the involvement of epigenetic modifications in cognitive functions (Di Francesco et al., 2015; Spiegel, Sewal & Rapp, 2014). For example, an inverse association was found between DNA methylation of mononuclear cells in peripheral blood and cognitive function of AD patients (Di Francesco et al., 2015). Epigenetic changes have also been found in the mice with post-stroke cognitive impairment (Wang et al., 2013; Wang et al., 2016). But there are few studies on exploring the relationship between epigenetic modifications and cholinergic system’s function in cognitive impairment after TBI.

Enriched environment (EE) intervention is a simple but valid neurorehabilitation strategy for improving cognitive impairment, which is widely used in the rehabilitation of a number of neurological diseases (Wang et al., 2016; Yu et al., 2014). Compared with standard environment (SE), EE can provide greater activity space, more communication with other members and more diversified stimulants (Yu et al., 2014). In animal models, EE has been shown to facilitate brain physiology and enhance recovery, which are associated with neuroplasticity (Wang et al., 2016; Yu et al., 2014). Studies have suggested that the role of EE is related to the reduction of oxidative damage or the restoration of hippocampal neurogenesis (Mármol et al., 2017; Speisman et al., 2013). It was also found that mice with TBI demonstrated a remarkable improvement in cognitive functions after exposure to EE (Bondi et al., 2014). However, the underlying mechanism of this improvement is still obscure.

Hence, in this study, we focused on the influence of EE intervention on the working memory of mice with TBI, and explored whether EE may affect the cholinergic system and epigenetic modifications of the prefrontal cortex.

## Methods and Materials

### Animals

All experimental procedures were carried out in accordance with the ARRIVE guidelines, and all experiments with mice were approved by the Animal Ethics Committee of Yangzhou University. Clean-grade mice (Species: Kunming, gender: male, age: postnatal week 6–8, body weight: 25–30 g) were provided by the Experimental Animal Center of Yangzhou University. All mice were housed under appropriate temperature (25 °C) and humidity (60%) in controlled atmosphere of 12 h–12 h light-dark cycle, and had free access to both water and food (Wang et al., 2016).

### Brain injury model

**Figure 1:**
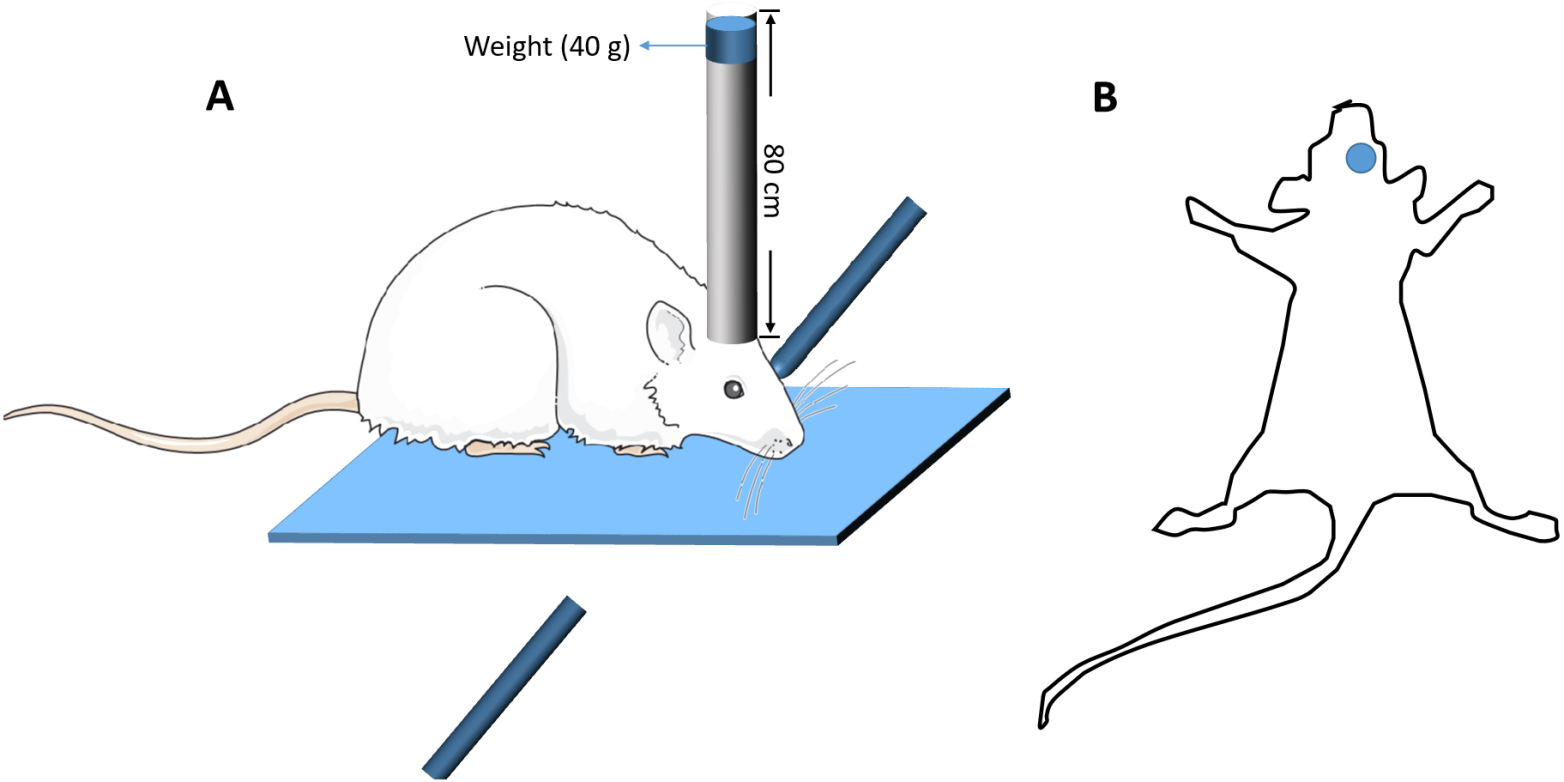
The diagrammatic figure of the mouse model of closed head injury. (A) The model device of brain injury (B) The site of brain injury.

The modified free-fall method was used to establish the mouse model of closed head injury (Kumar, Herbst & Strickland, 2012). Briefly, the device constitutes a vertical guide tube (13 mm inner diameter), dropping weights and a stereotactic frame. The mouse was anesthetized with an intraperitoneal injection of 0.15 ml 1% pentobarbital sodium, and the mouse head was fixed in a stereotactic frame. A 40 g weight from a height of 80 cm was freely fell down along a vertical tube, and struck the skull at the spot 2 mm lateral from the Bregma on the right (Fig. 1). A sponge was used to support the head to ensure no head rotation movement occurring during the impact, but some anteroposterior motion was allowed. Five minutes after the impact, the mice were put back into the cages for recovery. Sham-operated mice went through the same procedure, but the dropping weights procedure was removed. After surgery, all the mice including TBI mice and sham-operated mice were housed in the cages with standard environment for 14 days. Mice with TBI that could walk straight after 14 days, without bending towards one side, were screened for Y-maze test. Those TBI mice whose percentage of spontaneous alternations in the Y-maze test had significant difference compared with those of the sham-operated mice (*p* < 0.05) were identified as mice with working memory impairment and selected for further study (Wang et al., 2016). At the end, there were at least 60 TBI mice with working memory impairment.

### Y-maze test

**Figure 2:**
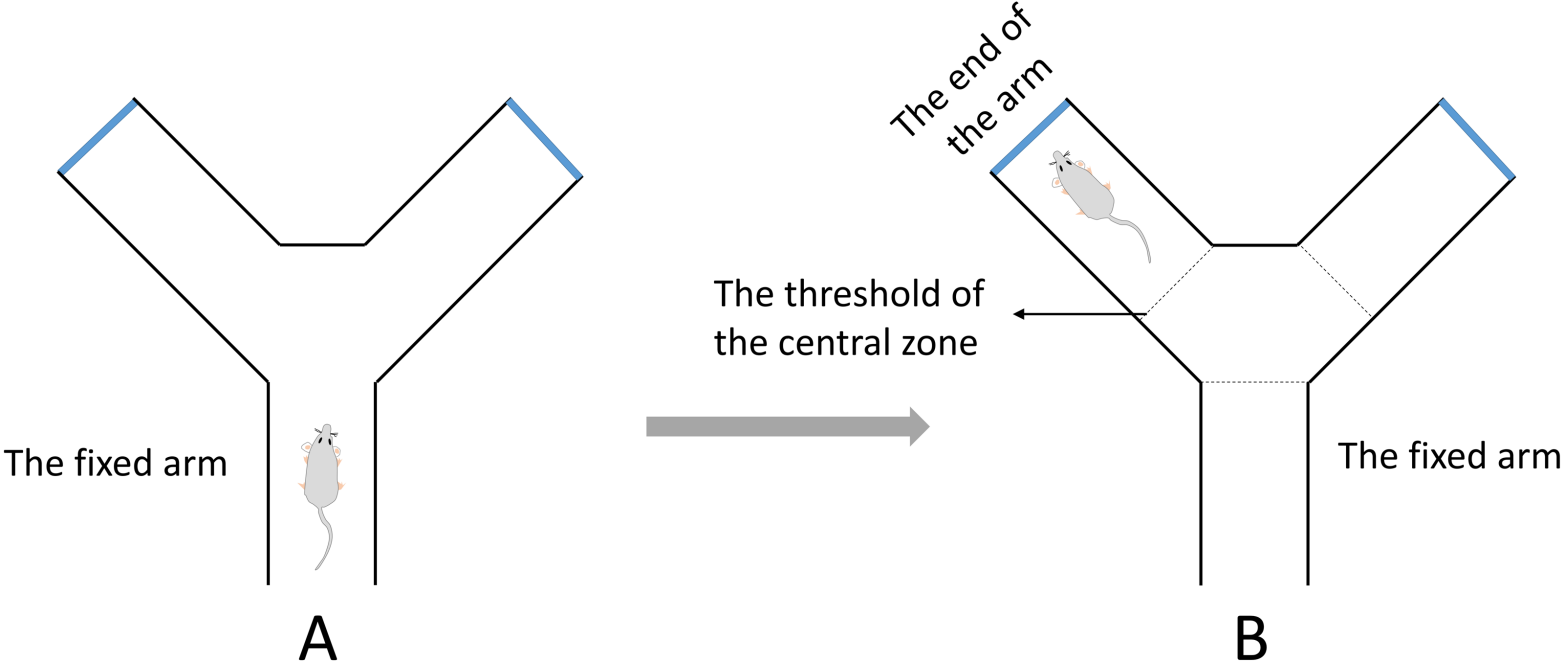
The diagrammatic figure of Y-Maze test. (A) During the test, the mouse was placed in the middle of the fixed arm and faced to the center of the maze. (B) One arm entry was defined as all four paws of the mouse crossed the threshold of the central zone and into the arm and the animal’s snout was oriented toward the end of the arm.

A Y-maze test for evaluating spatial working memory was performed in this study (Tucker, Fu & McCabe, 2016). The test was completed within a day. The apparatus was composed of three identical arms that was made up of plexiglass (30 cm long, 8 cm wide and 16 cm high) positioned at equal angles 120°. During the test, the mouse was placed in the middle of the fixed arm and faced to the center of the maze (Fig. 2A). Then the mouse was allowed to explore the maze freely for 8 min. One arm entry was defined as all 4 paws of the mouse crossed the threshold of the central zone and into the arm and the animal’s snout was oriented toward the end of the arm (Fig. 2B). A correct alternation was defined as the mouse that entered a different arm of the maze in each of 3 consecutive arm entries. The maximum number of alternations was equal to the sum of arms that the mouse entered minus two. The spontaneous alternation rate was calculated with the following formula: spontaneous alternation % = (number of correct alternations/number of maximum alternations) ×100%. The total number of arms entered was also recorded.

### Study design (Wang et al., 2016)

The selected TBI mice with working memory impairment were randomly assigned to 2 groups: TBI mice that lived in Standard Environment (TBI+SE), and TBI mice that lived in Enriched Environment (TBI+EE). Sham-operated mice still lived in Standard Environment. So the specific design of this study is as follows. Thirty TBI+SE mice and 30 sham-operated mice (SHAM+SE) were housed in standard cages (32 cm long ×20 cm wide ×13 cm high). Another 30 TBI+EE mice were caged in larger ones (60 cm long ×36 cm wide ×28 cm high) containing several shapes and colors of rodent toys and the running wheels. Toys were changed every four days regularly. The mice in different groups were housed in different environmental conditions (24 h a day) for 4 weeks before second Y-maze test .

### Brain sample preparation

The prefrontal cortex is the most related organ to working memory (Jimura et al., 2017; Sherwood et al., 2016). The left prefrontal cortex of mouse was selected for further analysis because the right frontal cortex was destroyed directly by TBI. After Y-maze test, all mice were sacrificed and the left prefrontal cortex was removed. After that, the brain tissues were quickly frozen in liquid nitrogen and stored at −80 °C for subsequent acetylcholine (Ach) level assessment and chromatin immunoprecipitation (ChIP) assay, as well as quantitative real-time polymerase chain reaction (Q-PCR), and western blotting analysis. Every 12 frozen left frontal cortex per group were used for western blotting analysis and every six frozen left frontal cortex per group were used for other different detection methods (Wang et al., 2016).

### Ach assay

Ach detection kit (Jiancheng, China) was used to detect the Ach levels (Wang et al., 2016) according to the manufacturer’s instructions. The concentration of Ach was calculated via the following equation: Ach concentration(_µ__g/mg protein_) =(OD_Sample_ − OD_Blankstandard_)∕(OD_Blankstandard_ − OD_Blank_) × 400 × *F*. *F* means the dilution factors of the sample protein concentrations, and OD refers to the optical density.

### RNA extraction and quantitative real-time PCR (Q-PCR)

Total RNA were extracted by using the RNA isolation kit (Promega, Madison, WI, USA) according to the manufacturer’s instructions. cDNA was synthesized from isolated RNA template by using avian myeloblastosis virus reverse transcriptase. The mRNA transcription of ChAT gene was detected and the primers used were as follows: forward primer (Akpan et al., 2008), (5′∼3′, AGC CCT GCT GTG ATC TTT GCT CG); reverse primer, (5′∼3′, CCT TGG CCC AGT CAG TGG GAA). Primers specific for GAPDH were used as control: forward primer (Wang et al., 2016), (5′∼3′, GTC ATA TTT CTC GTG GTT CAC ACC); reverse primer, (5′∼3′, CTG AGT ATG TCG TGG AGT CTA CTG G). The Q-PCR reaction conditions were as follows: 95 °C for 5 min, followed by 40 cycles of 95 °C for 45 s, 60 °C for 50 s, and 72 °C for 30 s. Q-PCR was performed by Sequence Detection System.

### Western blot assay

Proteins extracted from brain tissues (left prefrontal cortex of mouse) were separated via SDS-PAGE (Wang et al., 2016). Then the proteins were transferred onto nitrocellulose membranes and complexed with the following antibodies: ChAT (1:1,000, Merck Millipore), H3 (1:1,000, Bioworld), Ac-H3 (1:1,000, Bioworld), H4 (1:1,000, Bioworld), Ac-H4 (1:1,000, Bioworld) and CBP (1:1,000, Bioworld). Horseradish peroxidise (HRP)-conjugated anti-goat or anti-rabbit IgG antibodies (secondary antibody) were used to visualize the location of separated proteins via enhanced chemiluminescence. Finally, the membrane was exposed to film, and the blot intensity was quantified with densitometry. GAPDH was used as the loading control.

### Chromatin immunoprecipitation (ChIP) assay

Magna ChIP G tissue kit (Merck Millipore) was used in this study (Wang et al., 2016). During ChIP assay, prefrontal cortex was initially treated with tissue stabilization solution from the kit. Then, formaldehyde was used to cross-link the genomic DNA to chromatins. Subsequently, the compound was broken into 200–1,000 bp long fragments with a sonicator. After that, the chromatin lysate was incubated with rabbit anti mouse acetyl histone H3 antibody (1:1,000, Merck Millipore) or IgG (negative control) at 4 °C overnight under constant rotation. Subsequently, immune complex wash buffer was used to evaluate the cross-linked protein/DNA complexes and spin columns were used to purify the DNA. PCR was carried out with 40 cycles under the following reaction conditions: 94 °C for 20 s, 59 °C for 30 s, and 72 °C for 30 s, and the reaction volumes were set as 20 µl. Specific PCR primers were synthesized for amplifying target promoter sequences of ChAT gene (M promoter). The primer sequences were as follows: forward, 5′∼3′, CTGTGAGGAAGAGAGGCAGG; reverse, 5′∼3′, GACTGACTGCAAGCAAACCA (Aizawa & Yamamuro, 2010). After amplification, the agarose gel was used to separate the PCR products (10 µl) and then exposed to UV light. The light intensity was detected by densitometry to quantify the DNA content.

### Statistical analysis

The statistical data were analyzed by using SPSS 23.0 statistical software (SPSS, Chicago, IL, USA) and expressed as means ±  standard deviation (SD). Statistical differences among different groups were performed using one-way ANOVA followed by multiple-range test. *P* value of <0.05 was considered to be significant, and *p* < 0.01 was suggested to be extremely significant.

## Results

### The Y-maze test revealed the changes of spatial working memory of mice in different groups (*n* = 10)

**Figure 3:**
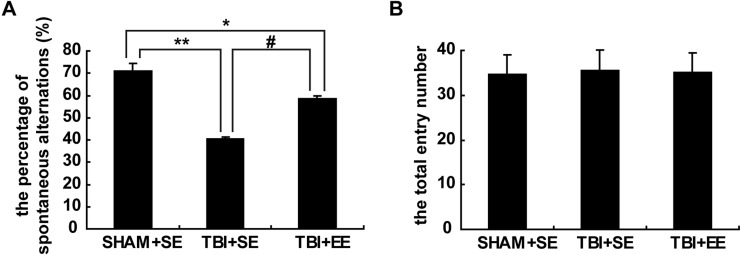
EE improves spatial working memory of mice with TBI. (A) The percentage of spontaneous alternations, (B) The total entry number of arms entry. (*) *p* < 0.05 compared with SHAM +SE; (**) *p* < 0.01 compared with SHAM+SE; (#) *p* < 0.05 compared with TBI+SE.

The result of the Y-maze test exhibited significantly lower percentage of spontaneous alternations of mice in the TBI+SE group (*p* < 0.01) as compared to that of the mice in the SHAM+SE group. After living in EE for 4 weeks, the mice in the TBI+EE group showed a significantly increased percentage of spontaneous alternations compared with the mice in TBI+SE (*p* < 0.05). However, the percentage of spontaneous alternations of TBI+EE group mice was still distinctly differentiated from that of SHAM+SE group mice (*p* < 0.05), (Fig. 3A). The total number of arms entered between the three groups showed no significant difference (*p* > 0.05), (Fig. 3B).

### EE improves cholinergic system’s function in the prefrontal cortex of contralateral side of TBI

#### Ach level in the prefrontal cortex (*n* = 6)

**Figure 4:**
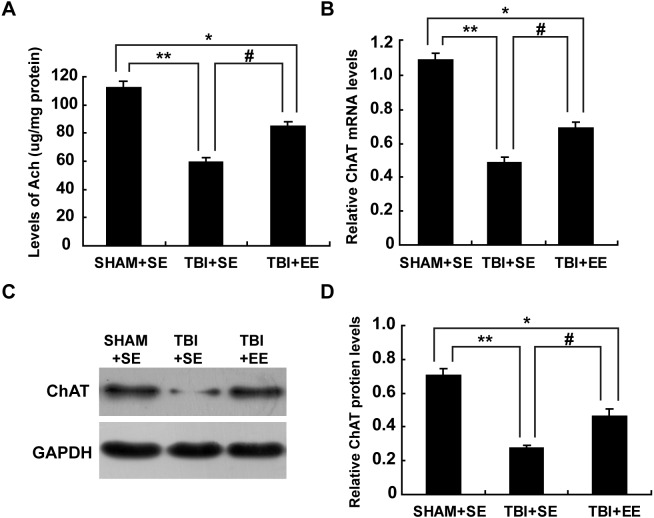
EE enhances cholinergic system’s function in the prefrontal cortex of contralateral side of TBI. (A) The changes of Ach levels of left prefrontal cortex of mice in different groups after EE; (B) The changes of mRNA levels of ChAT of left prefrontal cortex of mice in different groups after EE; (C) The changes of protein expression of ChAT of left prefrontal cortex of mice in different groups after EE; (D) Quantitative measurement of protein intensities in (C). (*) *p* < 0.05 compared with SHAM+SE; (**) *p* < 0.01 compared with SHAM+SE; (#) *p* < 0.05 compared with TBI+SE.

Ach is a critical indication of the function of cholinergic system in the brain (Wang et al., 2016). So, the changes in the Ach levels of the left prefrontal cortex (contralateral side of TBI) after EE intervention were examined. The TBI+SE group mice showed significantly decreased Ach levels in their left prefrontal cortex compared to those of the SHAM+SE group mice (*p* < 0.01). After living in EE for 4 weeks, the Ach levels of the TBI+EE group mice had a remarkable growth compared with those of the TBI+SE group mice (*p* < 0.05). However, the TBI+EE group mice still exhibited lower Ach levels than those of the SHAM+SE group mice (*p* < 0.05), (Fig. 4A).

#### mRNA levels and protein expression of ChAT in the prefrontal cortex (*n* = 6)

Ach is synthesized from acetyl coenzyme A (AcetylCoA) and choline by choline acetyl transferase (ChAT) (Nakauchi & Sumikawa, 2012). So, ChAT acts as another important marker for the function of cholinergic system. As seen from Fig. 4B, mRNA levels of ChAT in the left prefrontal cortex of the TBI+SE group mice were much lower than that of the SHAM+SE group mice (*p* < 0.01), (Fig. 4B). After living in EE for 4 weeks, mRNA levels of ChAT in the TBI+EE group mice were enhanced significantly compared with mRNA levels of ChAT in the TBI+SE group mice (*p* < 0.05). But still a significant difference in the mRNA levels of ChAT between the TBI+EE group mice and the SHAM+SE group mice were observed (*p* < 0.05), (Fig. 4B).

As shown in Figs. 4C and 4D, the changes of protein expression of ChAT of mice in different groups were in accordance with the changes of mRNA levels of ChAT of mice in different groups.

### EE enhances histone acetylation in the prefrontal cortex of contralateral side of TBI (*n* = 6)

**Figure 5:**
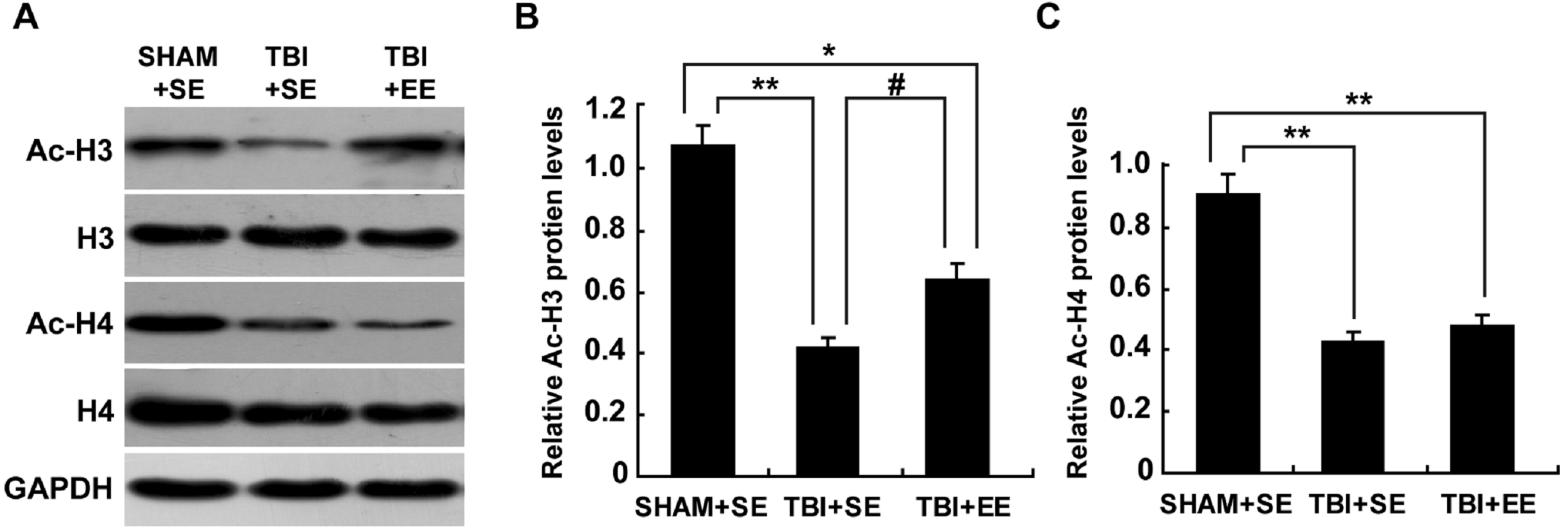
EE improves Ac-H3 protein expression in the prefrontal cortex of contralateral side of TBI. (A) The changes of Ac-H3 and Ac-H4 protein levels of left prefrontal cortex of mice in different groups after EE; (B) Statistics analysis on the changes of Ac-H3 protein levels of the left prefrontal cortex of mice in different groups after EE. (C) Statistics analysis on the changes of Ac-H4 protein levels of left the prefrontal cortex of mice in different groups after EE . (*) *p* < 0.05 compared with SHAM+SE; (**) *p* < 0.01 compared with SHAM+SE; (#) *p* < 0.05 compared with TBI +SE.

The levels of protein expression of Ac-H3 and Ac-H4 in the tissues are usually used to evaluate the changes in histone acetylation of the organism (Wang et al., 2013; Wang et al., 2016). As shown in Fig. 5, the protein expression of Ac-H3 and Ac-H4 of left prefrontal cortex of the TBI+SE group mice showed significant decrease compared to those of the SHAM+SE group mice (*P* < 0.01). After living in EE for 4 weeks, the Ac-H3 protein expression of the TBI+EE group mice exhibited a significant rise compared to that of the TBI mice living in the standard environment (*P* < 0.05). But no changes of Ac-H4 protein expression of the TBI+EE group mice compared with that of the TBI+SE group mice were observed (*P* > 0.05). The Ac-H3 protein expression of the TBI+EE group mice was still obviously different from that of the sham-operated mice living in the standard environment (*p* < 0.05).

### EE raises CREB binding protein (CBP) expression in the prefrontal cortex of contralateral side of TBI (*n* = 6)

**Figure 6:**
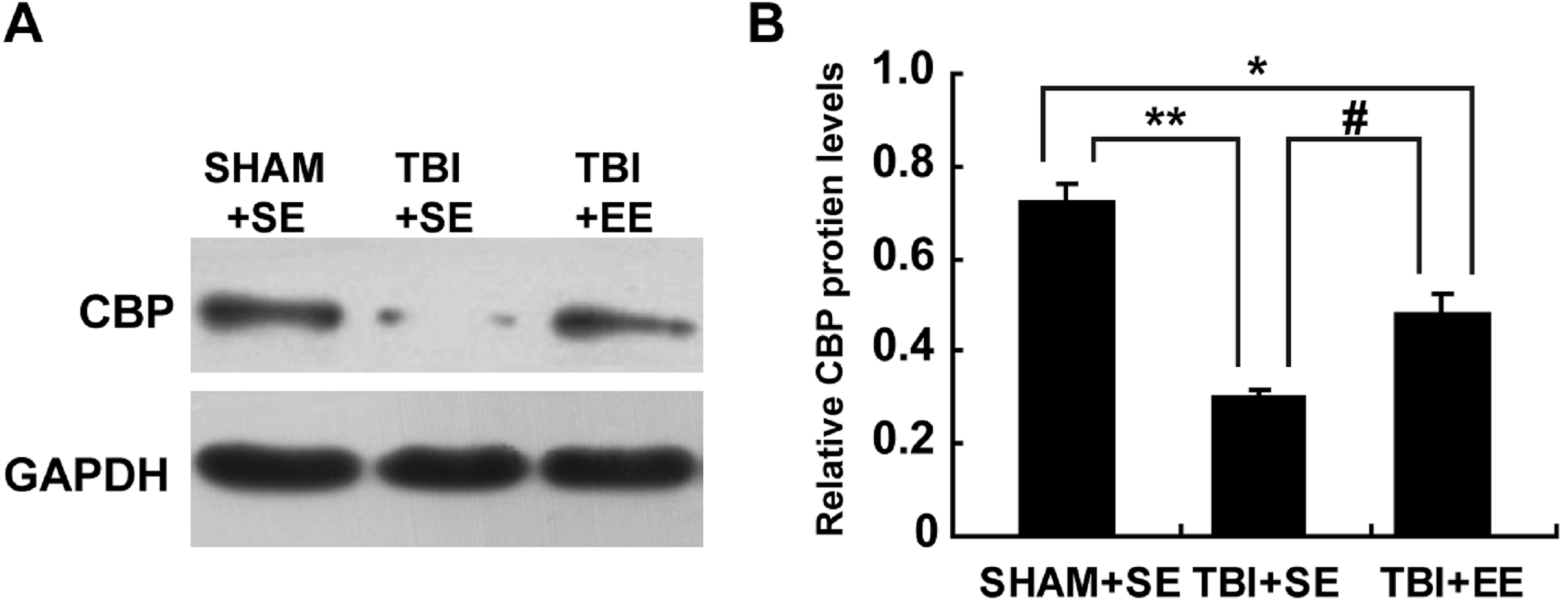
EE enhances CBP protein expression in the prefrontal cortex of contralateral side of TBI. (A) The changes of CBP protein levels of left prefrontal cortex of mice in different groups after EE; (B) Statistics analysis on the changes of CBP protein levels of the left prefrontal cortex of mice in different groups after EE. (*) *p* < 0.05 compared with SHAM+SE; (**) *p* < 0.01 compared with SHAM+SE; (#) *p* < 0.05 compared with TBI+SE.

Next, the changes of protein expression of CBP in the prefrontal cortex of contralateral side of TBI between different groups were observed. The expression levels of CBP in the TBI+SE group mice was decreased significantly compared to those of the SHAM+SE group mice (*p* < 0.01), (Fig. 6). The TBI+EE group mice showed significantly increased expression levels of CBP compared with those of TBI+SE group mice (*p* < 0.05). But still a significant reduction in the expression levels of CBP in the TBI+EE group mice compared to those of the SHAM+SE group mice was observed (*p* < 0.05), (Fig. 6).

### EE increases histone acetylation at ChAT gene promoter region in the prefrontal cortex of contralateral side of TBI (*n* = 6)

**Figure 7:**
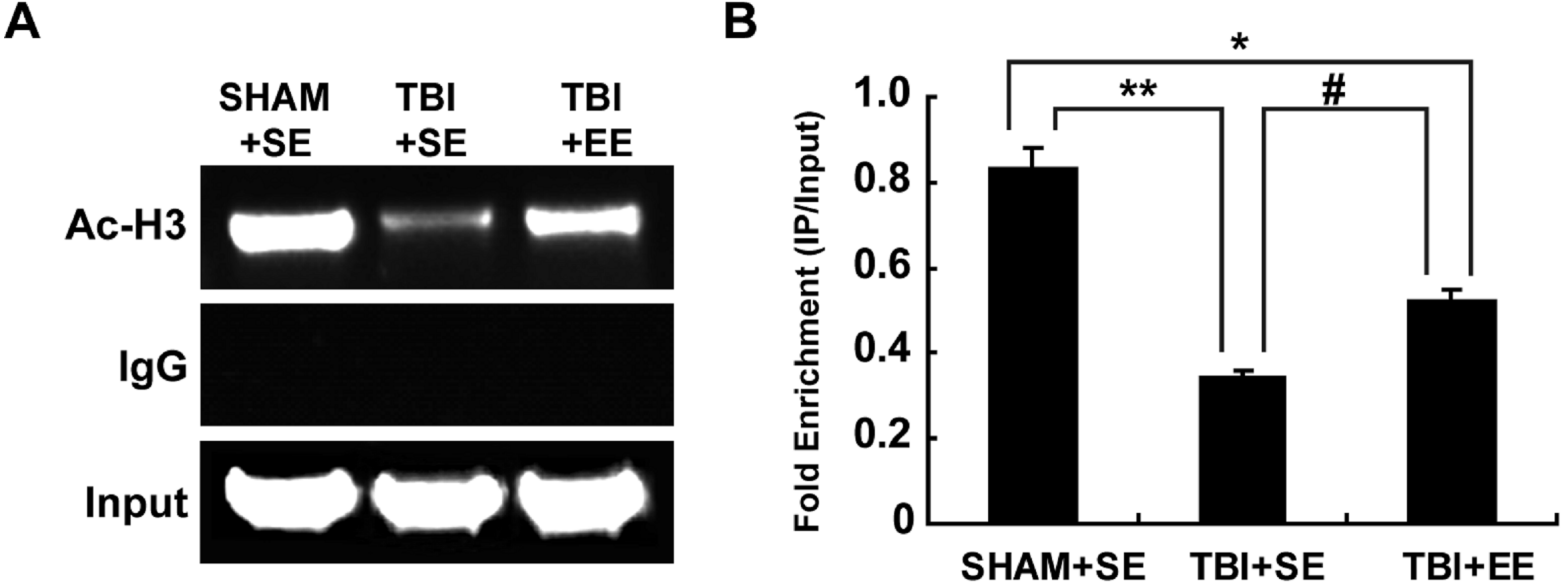
EE increases histone H3 acetylation at ChAT gene M-type promoter region in the prefrontal cortex of contralateral side of TBI. (A) The changes of histone H3 acetylation at ChAT gene M-type promoter region of left prefrontal cortex of mice in different groups after EE; (B) Statistics analysis on the changes of histone H3 acetylation at ChAT gene M-type promoter region of left prefrontal cortex of mice in different groups after EE (IP/Input). (*) *p* < 0.05 compared with SHAM+SE; (**) *p* < 0.01 compared with SHAM+SE; (#) *p* < 0.05 compared with TBI+SE.

According to the previous studies, the transcription of ChAT mRNA was involved in histone acetylation (Aizawa & Yamamuro, 2010; Aizawa, Teramoto & Yamamuro, 2012). Therefore, the changes of histone H3 acetylation of M-type promoter of ChAT gene in the prefrontal cortex of contralateral side of TBI were detected by chromatin immunoprecipitation assay. The results showed that the TBI+SE group mice exhibited an obvious reduction in histone H3 acetylation at ChAT gene M-type promoter region of the left prefrontal cortex compared to that of the sham-operated mice living in the standard environment (*p* < 0.01), (Fig. 7). After living in EE for 4 weeks, the levels of histone H3 acetylation at ChAT gene M-type promoter region of the prefrontal cortex in the TBI+EE group mice was increased remarkably compared to that of the TBI +SE group mice (*p* < 0.05). But a significant difference in the level of histone H3 acetylation at ChAT gene M-type promoter region of the TBI+EE group mice compared to that of the SHAM+SE group mice was still observed (*p* < 0.05) (Fig. 7).

## Discussion

At present, whether in developed countries or developing countries, TBI is a common neurological disorder. Many studies have shown that cognitive functions, such as working memory, are impaired after TBI (Dobryakova, Boukrina & Wylie, 2015; Zimmermann et al., 2017). Working memory impairment seriously affects the quality of life of patients. Acetylcholine is a neurotransmitter that has a significant influence on the working memory (Sun et al., 2017; Uzum et al., 2016 ). For example, it was found that the increase of Ach in the brain can improve the spatial working memory of rats (Uzum et al., 2016). ChAT catalyzes the synthesis of Ach and only exists in the cholinergic neurons. So, ChAT is considered as the best marker to demonstrate the function of cholinergic system (Wang et al., 2013; Wang et al., 2016). In this study, it was found that the Y-maze test performance of mice with traumatic right frontal lobe injury was significantly decreased compared to that of the mice without TBI. The Y-maze test is a widely used test for assessing the working memory (Tucker, Fu & McCabe, 2016). The Y-maze test results in this study showed that mice with TBI had impaired working memory. Meanwhile, the indexes of the cholinergic system’s function of the left prefrontal cortex of mice with TBI, including Ach level, and mRNA and protein expression of ChAT, also were significantly lower than those of mice without TBI. The cholinergic system’s function of right frontal lobe was also directly destroyed by TBI. Therefore, these results suggest that the decrease of the cholinergic system’s function in the frontal lobe leads to working memory impairment.

EE is one of the ways to improve cognitive dysfunction (Wang et al., 2016; Bondi et al., 2014). In this study, it was found that EE can improve the performance of Y-maze in mice with brain trauma. While this improvement of working memory may be related to the enhancement of cholinergic system’s function in the prefrontal cortex of contralateral side of TBI, including the improvement of the level of acetylcholine, acetyltransferase protein and mRNA.

We further explored the mechanism. Studies have found that histone acetylation is also associated with cognitive function (Wang et al., 2013; Lopez-Atalaya et al., 2011). Boosted histone acetylation can promote gene expression, while histone de-acetylation can restrain gene expression (Wang et al., 2016). The dynamic acetylation and deacetylation reactions are essential for maintaining homeostasis. The changes of Ac-H3 and Ac-H4 protein expression are usually used to estimate the acetylation homeostasis. It was found that the Ac-H3 and Ac-H4 protein expression in the prefrontal cortex of TBI mice were decreased remarkably compared with those of mice without TBI. These reflected that acetylation homeostasis in the prefrontal cortex of TBI mice was imbalanced, while some genes related with the working memory were restrained. After EE intervention, the Ac-H3 expression in the prefrontal cortex of TBI mice increased significantly. It meant EE can partially correct this imbalance of acetylation homeostasis.

CBP, an endogenous histone acetyl transferase, was considered to be very important for acetylation homeostasis (Wang et al., 2016; Lopez-Atalaya et al., 2011). In our study, the CBP level in the prefrontal cortex of contralateral side of TBI was decreased remarkably compared to that of mice without TBI, which was related to histone de-acetylation after TBI. Further studies showed that CBP may act as a molecular bridge to mediate the regulatory effect of EE in maintaining the balance of brain acetylation homeostasis, contributing to improved cognitive function (Wang et al., 2016; Lopez-Atalaya et al., 2011). After EE intervention, CBP-deficient (CBP+/-) mice showed much less significant enhancement of cognitive function and promotion of histone acetylation in the hippocampus compared with wild-type (CBP+/+) mice (Lopez-Atalaya et al., 2011). In our study, EE can enhance CBP level of TBI mice significantly. This suggested that the increase of CBP in the brain after EE intervention can improve the imbalance of acetylation homeostasis of the brain after TBI.

Previous studies showed that histone acetylation may affect the cholinergic system (Wang et al., 2016; Aizawa & Yamamuro, 2010). TSA, one of the histone deacetylases inhibitors, can promote histone acetylation, initiate ChAT mRNA transcription and enhance ChAT protein expression (Aizawa & Yamamuro, 2010). Our previous study also showed that icariin, a kind of traditional Chinese medicine monomer, can reverse the unbalanced acetylation homeostasis of the hippocampus after stroke, increase ChAT mRNA transcription and protein expression, and eventually increase the Ach levels of the hippocampus (Wang et al., 2013). TSA-induced ChAT gene transcription is associated with acetylated histone at ChAT gene promoter region (Aizawa & Yamamuro, 2010). According to Ac-H3 CHIP assay in this study, it was found that in the left prefrontal cortex of mice, the levels of acetylated histone H3 at ChAT gene promoter region were significantly decreased in TBI mice compared to that of the mice without TBI, while EE may significantly enhance the histone H3 acetylation of the same region in the ChAT gene promoter. These results suggested that histone acetylation exerted an influence over Ach synthesis.

Studies showed that CBP can combine the cAMP response elements (CREs) located in the gene enhancer and promoter regions to stimulate the acetylation of histone and promote the transcription of genes, including ChAT gene (Wang et al., 2016; Xie et al., 2014). In our study, we found that the increase of mRNA levels and protein expression of ChAT of TBI mice was consistent with the enhancement of acetylated histone H3 at ChAT gene promoter region and CREB binding protein after EE intervention. These results showed that EE can improve the working memory impairment through transcriptional regulation of ChAT gene by enhancing acetylated histone H3 of promoter region.

There is a limit in this study that the TBI was made when a mouse was anesthetized, which might be different from TBI made when a mouse was awake. Also, more clinical implications based on the study results will be helpful.

## Conclusion

In conclusion, the evidences provided by this study are as follows:

1. TBI causes working memory impairment, which is correlated to decreased acetylcholine and choline acetyl transferase in the prefrontal cortex.
2. Histone acetylation in the prefrontal cortex of the contralateral side of TBI is decreased. Besides, acetylated histone H3 at ChAT gene promoter region and CREB binding protein were also declined.
3. EE can improve the working memory impairment of TBI mice, enhance the function of cholinergic system and increase histone acetylation of the prefrontal cortex of contralateral side of TBI, which are associated with the effect of EE that can reverse the decrease of CREB binding protein expression and enhance acetylated histone H3 at ChAT gene promoter region in the prefrontal cortex of contralateral side of TBI.

##  Supplemental Information

10.7717/peerj.6113/supp-1 Table S1 The result of Y-maze test Ten mice were randomly selected from each group and tested for Y-maze Test. The result of the Y-maze test exhibited significantly lower percentage of spontaneous alternations of mice in the TBI+SE group (*p* < 0.01) as compared to that of the mice in the SHAM+SE group. After 4 weeks living in EE, compared with the TBI+SE group mice, the TBI+SE group mice showed a significantly increased percentage of spontaneous alternations (*p* < 0.05). However, the percentage of spontaneous alternations of TBI+EE group mice was still distinctly different from that of SHAM+SE group mice (*p* < 0.05). The total number of arms entered between three groups has no significant difference (*p* > 0.05).

10.7717/peerj.6113/supp-2 Table S2 The result of the cholinergic system’s function Six left prefrontal cortex of mice per group were used to detect cholinergic system’s function. The TBI+SE group mice showed significantly decreased Ach levels in their left prefrontal cortex compared to those of the SHAM+SE group mice (*p* < 0.01). After living in EE for 4 weeks, the Ach levels of the TBI+EE group mice had a remarkable growth compared with those of the TBI+SE group mice (*p* < 0.05). However, the TBI+EE group mice still exhibited lower Ach levels than those of the SHAM+SE group mice (*p* < 0.05). The changes of protein expression and mRNA levels of ChAT of mice in different groups were in accordance with the changes of Ach levels of mice in different groups.

10.7717/peerj.6113/supp-3 Table S3 The result of histone acetylation in the prefrontal cortex of contralateral side of TBI The result of histone acetylation in the prefrontal cortex of contralateral side of TBI. The levels of protein expression of Ac-H3 and Ac-H4 were examined. The left prefrontal cortex’s Ac-H3 and Ac-H4 protein expression of the TBI+SE group mice had significant decrease in comparison with those of the SHAM+SE group mice (*P* < 0.01). After for 4 weeks living in EE, the left prefrontal cortex’s Ac-H3 protein expression of the TBI+EE group mice exhibited a significant rise in comparison with that of the TBI mice living in the standard environment (*P* < 0.05). But there had no changes of Ac-H4 protein expression of the TBI+EE group mice compared with that of the TBI+SE group mice (*P* > 0.05). The left prefrontal cortex’s Ac-H3 protein expression of the TBI+EE group mice was still obviously different from that of the sham-operated mice living in the standard environment (*P* < 0.05).

10.7717/peerj.6113/supp-4 Table S4 The result of CREB binding protein (CBP) expression in the prefrontal cortex of contralateral side of TBI It was also observed the changes of protein expression of CBP in the prefrontal cortex of contralateral side of TBI between different groups. The expression levels of CBP in the TBI+SE group mice was decreased significantly compared with those of the SHAM+SE group mice (*p* < 0.01). The TBI+EE group mice showed significantly increased expression levels of CBP compared with the TBI+SE group mice (*p* < 0.05). But there was still a significant reduction in expression levels of CBP in the TBI+EE group mice in comparison with that of the SHAM+SE group mice (*p* < 0.05).

10.7717/peerj.6113/supp-5 Table S5 The result of histone acetylation at ChAT gene promoter region in the prefrontal cortex of contralateral side of TBI The changes of histone H3 acetylation of M-type promoter of ChAT gene in the prefrontal cortex of contralateral side of TBI were detected by chromatin immunoprecipitation assay.As showed in the table, the TBI+SE group mice exhibited an obvious reduction in histone H3 acetylation at ChAT gene M-type promoter region of the left prefrontal cortex in comparison with that of the TBI mice living in the standard environment (*P* < 0.01). After living for 4 weeks in EE, the level of histone H3 acetylation at ChAT gene M-type promoter region of the prefrontal cortex in the TBI +EE group mice was increased remarkably compared with that of the TBI+SE group mice (*p* < 0.05). But there was still a significant difference in the level of histone H3 acetylation at ChAT gene M-type promoter region of the TBI+EE group mice in comparison with that of the SHAM+SE group mice (*p* < 0.05).

10.7717/peerj.6113/supp-6 Supplemental Information 1 Full-length uncropped blots (Figs. 2–5).

## Additional Information and Declarations

### Data Availability

The following information was supplied regarding data availability: The raw data are available as Supplemental Files.
